# Effects of tolcapone and bromocriptine on cognitive stability and flexibility

**DOI:** 10.1007/s00213-018-4845-4

**Published:** 2018-02-09

**Authors:** Ian G. M. Cameron, Deanna L. Wallace, Ahmad Al-Zughoul, Andrew S. Kayser, Mark D’Esposito

**Affiliations:** 1 Donders Institute for Brain, Cognition and Behaviour, Centre for Cognitive Neuroimaging, Radboud University Nijmegen, Nijmegen, The Netherlands; 2 Department of Neurological Surgery, University of California, San Francisco, San Francisco, CA USA; 3 Helen Wills Neuroscience Institute, University of California, Berkeley, Berkeley, CA USA; 4 Department of Neurology, University of California, San Francisco, San Francisco, CA USA; 5 Department of Neurology, VA Northern California Health Care System, Martinez, CA USA; 6 Department of Psychology, University of California, Berkeley, Berkeley, CA USA

**Keywords:** Saccade, Dopamine, Prefrontal cortex, Basal ganglia, Tolcapone, Bromocriptine

## Abstract

**Rationale:**

The prefrontal cortex (PFC) and basal ganglia (BG) have been associated with cognitive stability and cognitive flexibility, respectively. We hypothesized that increasing PFC dopamine tone by administering tolcapone (a catechol-O-methyltransferase (COMT) inhibitor) to human subjects should promote stability; conversely, increasing BG dopamine tone by administering bromocriptine (a D2 receptor agonist) should promote flexibility.

**Objective:**

We assessed these hypotheses by administering tolcapone, bromocriptine, and a placebo to healthy subjects who performed a saccadic eye movement task requiring stability and flexibility.

**Methods:**

We used a randomized, double-blind, within-subject design that was counterbalanced across drug administration sessions. In each session, subjects were cued to prepare for a pro-saccade (look towards a visual stimulus) or anti-saccade (look away) on every trial. On 60% of the trials, subjects were instructed to switch the response already in preparation. We hypothesized that flexibility would be required on switch trials, whereas stability would be required on non-switch trials. The primary measure of performance was efficiency (the percentage correct divided by reaction time for each trial type).

**Results:**

Subjects were significantly less efficient across all trial types under tolcapone, and there were no significant effects of bromocriptine. After grouping subjects based on Val158Met COMT polymorphism, we found that Met/Met and Val/Met subjects (greater PFC dopamine) were less efficient compared to Val/Val subjects.

**Conclusions:**

Optimal behavior was based on obeying the environmental stimuli, and we found reduced efficiency with greater PFC dopamine tone. We suggest that greater PFC dopamine interfered with the ability to flexibly follow the environment.

## Introduction

Among the myriad of processes that constitute “cognitive control” (Miller and Cohen 2001; Fuster 2001; Cools and D’Esposito 2011; Stuss 2011), two opposing processes governing behavior have been described: cognitive *stability* and cognitive *flexibility*. Cognitive stability refers to the ability to establish and maintain a “task set,” so that goal-directed behaviors can be executed despite interference from conflicting alternative behaviors (Sakai 2008). This ability is thought to be critically dependent upon the function of the prefrontal cortex (PFC), which is known to be important to working memory, rule representation, and resistance to distraction (Miller and Cohen 2001; Fuster 2001; Cools and D’Esposito 2011). In contrast, cognitive flexibility refers to the ability to override a task set in order to perform an alternative behavior (Robbins 2007), and this ability is thought to be mediated by circuits involving the basal ganglia (BG) and the PFC (Cools and D’Esposito 2011). While the BG do not operate in isolation (as cortical sensory, motor, and association areas project to the striatum (Gerfen and Surmeier 2011; Shipp 2017)), BG circuits may have a particular role in assisting in the flexible switching between responses (Robbins 2007).

Both stability and flexibility can be influenced significantly by neuromodulators acting in the PFC and BG: dopamine, for example, is a well-known modulator of cortico-basal ganglia networks (Cools and D’Esposito 2009; Gerfen and Surmeier 2011), and it may differentially impact stability and flexibility through its effects on different dopamine receptor subtypes (Seamans and Yang 2004). In the PFC, dopamine D1 receptor stimulation is thought to support the maintenance of information, by promoting recurrent activity in PFC networks (thereby facilitating stability), whereas D2 receptor stimulation is thought to support flexibly switching between different representations of information, by increasing the responsiveness to new inputs (Durstewitz and Seamans 2008). D1 receptors are thought to be more dominant than D2 receptors in the PFC, however (Cools 2006; Cools and D’Esposito 2011), suggesting that if dopamine tone is increased in PFC, there would be a net increase in stability.

In comparison, the basal ganglia contains both D1 and D2 receptors on striatal medium spiny neurons, with D1 receptors found predominantly on those neurons constituting the “direct” pathway, and D2 receptors are found predominantly on these neurons constituting the “indirect” pathway (Surmeier et al. 2007; Gerfen and Surmeier 2011). Models of these direct (facilitatory) and indirect/hyperdirect (inhibitory) pathways in the BG provide a sensible mechanism to explain how the brain can override one response with an alternative (Mink 1996; Nambu 2008). A well-known model of BG function proposes that the direct pathway facilitates cortical signals important for producing the desired behavior, while the indirect pathway aids in inhibiting competing cortical signals, and that the net action of dopamine is to boost the facilitatory effect of the direct pathway (D1) and reduce the inhibitory effect of the indirect pathway (D2) (Mink 1996; Surmeier et al. 2007; Nambu 2008; Gerfen and Surmeier 2011). This model posits that dopamine excites striatal neurons in the direct pathway through D1 receptors, while it inhibits striatal neurons in the indirect pathway through D2 receptors (Nambu 2008). The net result from this dual action of dopamine would be the facilitation of a desired behavior, but with the consequence that there would be reduced suppression of other behaviors, promoting flexible switching to an alternative action.

Here, we test hypotheses about the relative roles of dopamine in the PFC and basal ganglia with respect to cognitive stability and flexibility during performance of a saccadic eye movement task, where dopamine tone was manipulated pharmacologically. We hypothesized that augmenting dopamine tone in PFC in human subjects would favor D1 stimulation, and hence, promote cognitive stability (Cools and D’Esposito 2011; Frank and Fossella 2011). In contrast, we hypothesized that D2 stimulation (Cools 2006; Kvernmo et al. 2006; Cools et al. 2007) would promote cognitive flexibility, primarily by a basal ganglia mechanism.

To test our first hypothesis, we administered tolcapone, a catechol-O-methyltransferase (COMT) inhibitor that preferentially increases dopamine tone in the PFC because COMT has a greater role in dopamine metabolism in the PFC compared to the basal ganglia (Männistö and Kaakkola 1999; Tunbridge et al. 2004). (We note that tolcapone could have effects beyond PFC—for example, in the hippocampus (Laatikainen et al. 2013)—but the important comparison here is its relative action in the PFC compared to the BG). Additionally, to evaluate a secondary and independent correlate of COMT activity, we divided subjects based on the functional Val158Met COMT polymorphism to assess differences in behavior depending on relative COMT activity between these groups of individuals. The Val allele results in greater COMT activity, and hence greater degradation of dopamine compared to the Met allele (Lachman et al. 1996; Chen et al. 2004).

To test our second hypothesis, we administered the dopamine D2 agonist bromocriptine (Kimberg et al. 1997; Cools 2006; Kvernmo et al. 2006; Cools et al. 2007). While systemic bromocriptine would have effects on flexibility in both the PFC and BG, D2 receptors are found in relatively higher concentration in the BG (Cools 2006); thus, we hypothesized that bromocriptine would have a greater effect on flexibility via action on the indirect pathway of the BG.

Under the influence of tolcapone, bromocriptine, or placebo, subjects performed an interleaved pro-/anti-saccade task that was designed to require both cognitive stability and cognitive flexibility and was hypothesized to recruit PFC and BG circuits accordingly. To perform a pro-saccade, subjects simply look towards a peripheral stimulus, but to perform an anti-saccade, subjects must execute a voluntary eye movement in the direction opposite to the peripheral stimulus (Hallett 1978; Munoz and Everling 2004). Previous lesion, human neuroimaging, and physiology studies have shown that maintenance of an anti-saccade task set depends critically upon the dorsolateral PFC (Pierrot-Deseilligny et al. 2005; Everling and Johnston 2013). Saccade programming also involves the direct and indirect pathways of the BG (Hikosaka et al. 2000; Watanabe and Munoz 2011), and people with Parkinson’s disease (PD), a primarily BG disorder, show anti-saccade deficits (Chan et al. 2005; Hood et al. 2007). An interleaved pro-/anti-saccade also demands flexibility, due to the fact that subjects must establish the appropriate task set (pro- or anti-) on a trial-by-trial basis, based on an external cue (Munoz and Everling 2004). Finally, efficient performance of pro- and anti-saccades has been demonstrated to be sensitive to dopamine (Hood et al. 2007; Watanabe and Munoz 2011; Cameron et al. 2012).

Here, we utilized a particular variant of the pro-/anti-saccade task to further evoke flexibility in terms of overriding a prepared behavior with an alternative (Cameron et al. 2007): subjects are sometimes required to switch suddenly from a pro (look towards)-saccade to an anti-saccade or from an anti-saccade to a pro-saccade. Thus, on switch trials, one task set must be inhibited, while another is initiated. We hypothesized that switching behavioral responses in this fashion more explicitly recruits the BG, and have confirmed previously using functional MRI, and by studying patients with Parkinson’s disease, that the BG are important to this form of flexibility (Cameron et al. 2009, 2010).

Taken together, we predicted that tolcapone would promote stability, so would improve performance on non-switch trials, particularly anti-saccades. Additionally, we hypothesized tolcapone would have greater effects at improving performance on such trials in subjects with the Val allele. In contrast, bromocriptine would promote flexibility, by acting on D2 receptors of the BG, so as to facilitate performance on switch trials.

## Methods

### Subjects

All subjects gave written informed consent in accordance with the Committee for the Protection of Human Subjects at the University of California, San Francisco and University of California, Berkeley. Subjects first underwent a history and physical exam, as well as blood testing for liver function and urine screening for drugs of abuse, to ensure that there were no medical contraindications to tolcapone or bromocriptine use. We ultimately invited 22 subjects to participate in the saccade task, and 19 agreed to participate and were able to complete all 3 days. However, due to problems with poor eye-tracking quality in some of the subjects on some days, a final total of 16 subjects provided data from all three sessions. These 16 subjects were in the age range of 21–36 (mean = 24), were all right handed, and 11 were male.

### Medications

Subjects were randomized in a double-blind, counterbalanced, placebo-controlled fashion to receive placebo, a single 1.25 mg dose of bromocriptine, or a single 200 mg dose of tolcapone on their first visit. The remaining treatments were administered on their second and third visits, respectively, such that every subject received each treatment on one of the three study days. Because tolcapone can discolor the urine, and therefore can potentially un-blind subjects, each capsule was compounded with 25 mg of the B-vitamin riboflavin in order to mask this effect. On each day, subjects were given the drug in the morning. Subjects were then monitored throughout the day, during which time they also performed a series of cognitive tasks (unrelated to the present study, including a food choice task and a delay discounting task) but always ending with the saccade task presented here. Each subject began the saccade task at approximately 5 h post drug administration and completed it by 6 h. Both dopaminergic drugs are expected to have pharmacodynamically relevant serum concentrations for greater than 6 h (Dingemanse et al. 1994; Nyholm 2006; Kvernmo et al. 2006).

### Saccade task

We utilized a particular variant of a pro-/anti-saccade task requiring subjects to switch suddenly from a pro-saccade to an anti-saccade, or from an anti-saccade to a pro-saccade, on a subset of trials (Cameron et al. 2007). We hypothesized that switching a saccade response in this fashion more explicitly recruits the facilitatory (direct) and inhibitory (indirect) pathways of the BG and have confirmed using functional MRI in healthy subjects and patients with Parkinson’s disease that the basal ganglia are critical for this form of flexibility (Cameron et al. 2009, 2010).

**Fig. 1:**
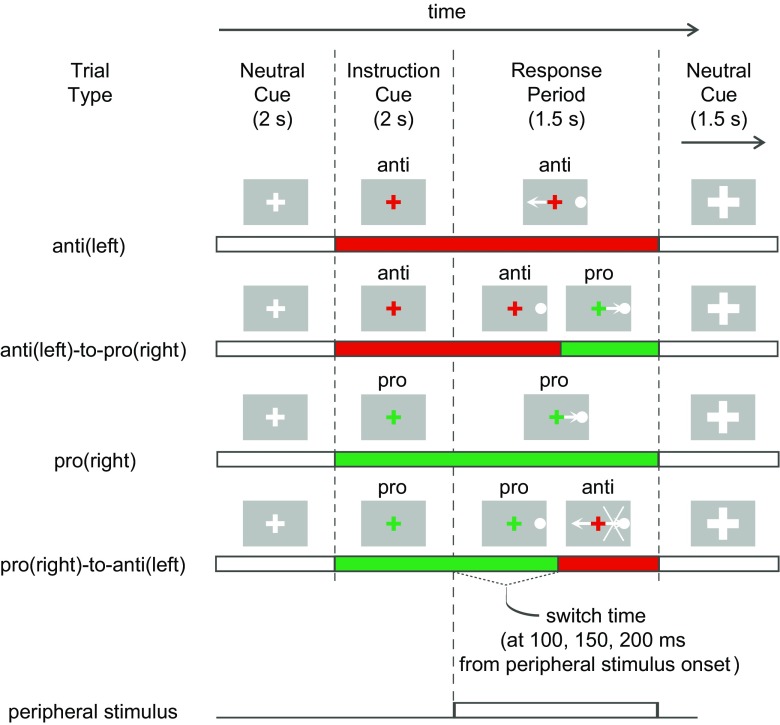
Paradigm and timing of trial events. Shown are examples of four trial types in which the peripheral stimulus was on the right side. On switch trials, the switch to the opposite task could occur at 100, 150, or 200 ms following peripheral stimulus onset, and subjects were instructed to respond to the final instruction only

Subjects performed 12 blocks of 20 trials of a pro-/anti-saccade task on each day. A neutral cross cue (2 s) was presented. Subsequently, a green or red cross (2 s) was presented instructing subjects to adopt a pro-saccade (look towards) or anti-saccade (look away) task set (Fig. 1). Then, a peripheral stimulus (a blue circle of 1 degree of visual angle) appeared at 12° pseudorandomly to the left or right for 1.5 s. On 60% of the trials, the instruction switched to the opposite color at 100, 150, or 200 ms after peripheral stimulus onset. These switch times were chosen because previous evidence suggested they are in the critical time for producing switch costs, indicating that an initial response program had to be switched (Cameron et al. 2007, 2009). The 60% switch frequency was chosen because we did not want non-switch trials to be asymmetrically easier to perform than switch trials, and we also wanted to increase the switch trial frequency to have sufficient power to examine differences across the switch times. Importantly, our previous study showed that switch costs are produced with switch trials up to 75% in probability, demonstrating that predictability cannot override the tendency to prepare the instructed response, resulting in a cost for switching (Cameron et al. 2007). Subjects were told to be quick, but accurate, and to obey the final instruction color. Another neutral cross (1.5 s) marked the return to fixation and ended each trial.

### Eye tracking and stimuli

An Eyelink 1000 (SR Research) system was used to track the right eye at a sample rate of 500 Hz. A CRT monitor with a 1024 × 768 resolution was used to present the visual targets, and the task was programmed in MATLAB 2011b using Psychtoolbox.

### Analysis

Our primary analysis of interest was “efficiency,” which is a combined measure of accuracy (i.e., correct direction (%)) divided by reaction time (ms) (Machizawa and Driver 2011). However, we also analyzed reaction times and accuracies separately.

A linear mixed effects model was performed in R 3.1.2, treating efficiency (or reaction time or accuracy) as the dependent variable, with initial task (pro or anti) and switch condition (non-switch or switch) as fixed effects. For this analysis, we collapsed across the three different possible onset times for the switch command. We accounted for possibly different effects for the drugs on a given subject, by treating the subject as a random effect and drug (bromocriptine, placebo, tolcapone) as a random slope. Post hoc multiple comparisons of means were employed using the Tukey’s method, after performing an analysis of deviance F-test on the results of the mixed-effects model in R.

We performed three secondary analyses on the saccade behaviors as follows. First, we examined the effects of the COMT genotype (Val/Val *N* = 4; Val/Met, *N* = 7; Met/Met *N* = 5) (collapsed across Switch Time) on efficiency. Second, we examined the effects of Switch Time (100, 150, 200 ms) on efficiency as a fixed effect in the above regression analysis but using only the switch trials. Finally, to explore whether the drugs influenced vigilance (the ability to maintain attention), we divided the runs into the first and second halves, adding the factor half to the initial task × switch time × drug models for efficiency, reaction times, and accuracy separately. (Changes in vigilance can be assessed by performance decrements in reaction time and/or accuracy as the time-on-task increases (Lim et al. 2012)).

In addition to this method to explore vigilance effects, we also examined performance (number of correct answers completed in 90 s) on the pen and paper version of the Digit Symbol Substitution Test (DSST), a component of the Wechsler Adult Intelligence Scale. The DSST measures sustained attention, response speed, visuomotor coordination, and set shifting (Lezak et al. 2004), so we used this test as a general measure of motor and cognitive functions to complement the saccade task. The DSST was performed prior to the saccade task (approximately 4–5 h after drug administration).

## Results

For performance efficiency, there was a main effect of drug (*F*(2, 35.6) = 4.00, *P* < 0.05), initial task (Pro or Anti; *F*(1, 149.3) = 8.29, *P* < 0.01), and switch condition (Switch or Non-switch; *F*(1, 149.3) = 82.62, *P* < 0.001). There was also an initial task × switch condition interaction (*F*(1, 149.3) = 22.67, *P* < 0.001), but no other interactions were significant (all *P*’s > 0.21).

**Fig. 2:**
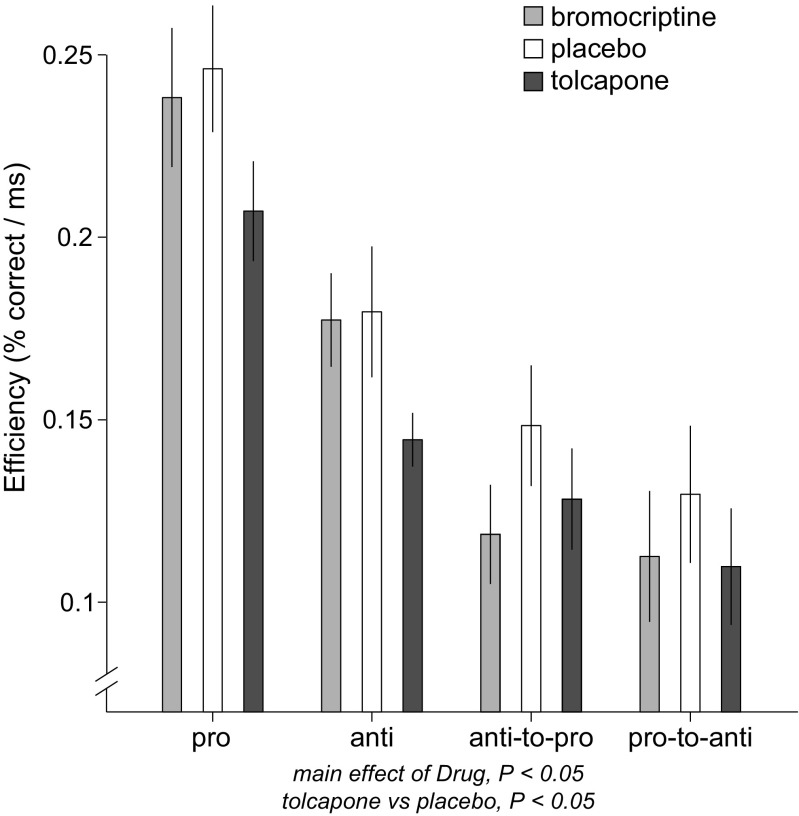
Effects of drug (bromocriptine, light gray shading; placebo, white shading; or tolcapone, dark gray shading) on efficiency (percent correct / reaction time) grouped according to the four trial types. Trial types were collapsed across direction (left or right) and switch time (100, 150, or 200 ms) on the switch trials (anti-to-pro and pro-to-anti). Significant results from the mixed-effects model or post hoc tests involving drug are indicated, revealing reduced efficiency under tolcapone

These results demonstrate that drug influenced performance efficiency across all trial types (Fig. 2), such that tolcapone reduced efficiency as compared to placebo (*z* = − 2.85, *P* < 0.05), but efficiency did not differ between bromocriptine and placebo (*z* = − 1.42, *P* = 0.32). The main effects and interactions involving initial task and switch condition are consistent with previous observations with this task (Cameron et al. 2007, 2009), such that performing an anti-saccade is less efficient (greater latency, increased direction errors) than performing a pro-saccade, switch trials are less efficient than non-switch trials (especially during switches from a pro to an anti-saccade), and efficiency is highest for non-switch pro-saccades.

**Fig. 3:**
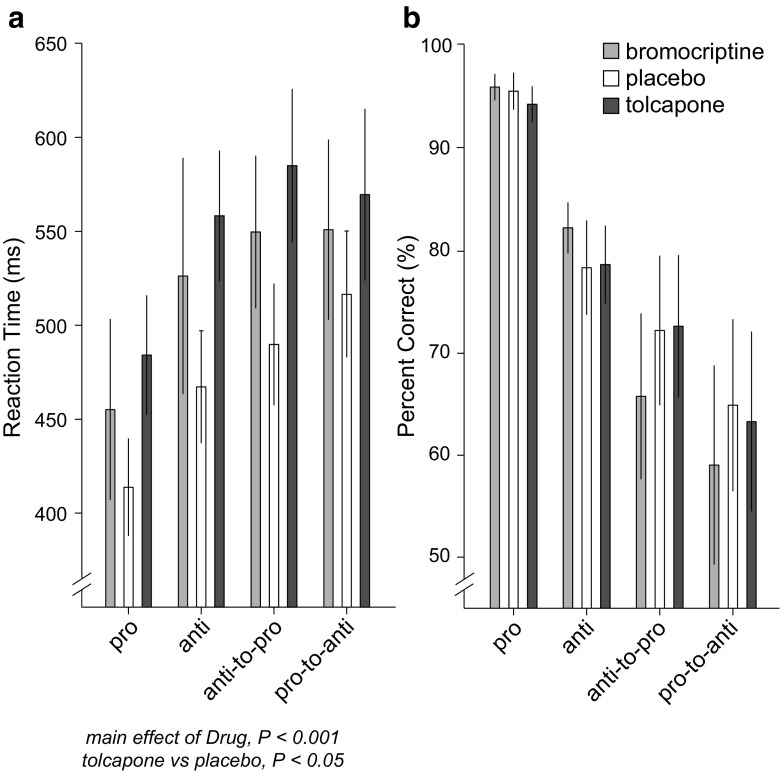
Effects of drug on reaction time (**a**) and percent correct (**b**), displayed as in Fig. 2. Significant results from the mixed-effects model or post hoc tests involving drug are indicated, revealing increased reaction times under tolcapone

Next, we assessed drug effects on reaction times and accuracy (i.e., percentage correct direction) separately. For saccade reaction time (Fig. 3a), there was a significant main effect of drug, *F*(2, 10.7) = 35.65, *P* < 0.001, as subjects were slower under tolcapone compared to placebo, *z* = 2.58, *P* < 0.05, but not slower under bromocriptine compared to placebo (*z* = 1.57, *P* = 0.20). There were also significant main effects of initial task, *F*(1, 149.0) = 6.38, *P* < 0.05, and switch condition, *F*(1, 149.0) = 21.24, *P* < 0.001, and a significant initial task by switch condition interaction, *F*(1, 149.0) = 7.25, *P* < 0.001. In summary, the results with reaction time mirror the results with efficiency, such that anti-saccades are performed more slowly than pro-saccades, switch trials are slower than non-switch trials, and reaction times are fastest for non-switch pro-saccades.

In comparison, there were no significant effects of drug on accuracy, *P* > 0.13 (Fig. 3b), but there was a main effect of switch condition, *F*(1, 150.0) = 67.53, *P* < 0.001, and a significant initial task by switch condition interaction, *F*(1, 150.0) = 20.44, *P* < 0.001. This finding shows that accuracy on the tasks was affected in a predictable fashion (i.e., worse accuracy on anti-saccades and switch trials, such that pro-to-anti-saccade switch trials were performed with the worst accuracy (Fig. 3b)).

**Fig. 4:**
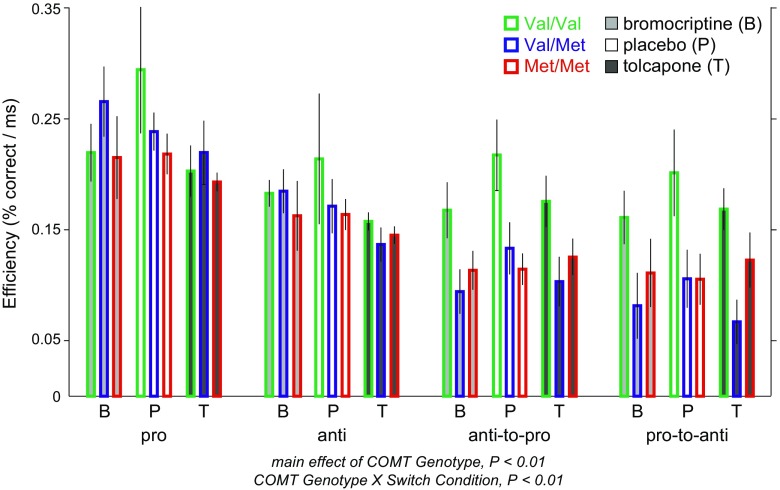
Effects of COMT Val/Met polymorphism (Val/Val, green border; Val/Met, blue border; Met/Met, red border) in addition to drug on efficiency. Bars are grouped for the four trial types and according to drug (B = bromocriptine, light gray shading; P = placebo, white shading; T = tolcapone, dark gray shading). Significant results from the mixed-effects model or post hoc tests involving COMT genotype are indicated, indicating that Val/Val subjects were more efficient, and that Val/Val subjects showed smaller performance differences between non-switch (pro, anti) and switch (anti-to-pro, pro-to-anti) trial types

To further explore our finding of a main effect of drug on performance efficiency, we divided subjects based on COMT genotype (Val/Val, Val/Met, Met/Met). We found a main effect of COMT genotype (*F*(2, 13.0) = 5.16, *P* < 0.05; Fig. 4); Val/Val subjects were more efficient than Val/Met subjects (*z* = 2.93, *P* < 0.01) and Met/Met subjects (*z* = 2.80, *P* < 0.05). There was also a significant COMT by switch condition interaction (*F*(2, 129.2) = 7.02, *P* < 0.001, as Val/Val subjects showed smaller performance differences between switch and non-switch trials. However, there was no COMT by drug interaction (*P* = 0.24).

Additional secondary analyses were performed to ensure that the task was being performed as expected. We found a main effect of switch time, *F*(2, 224.1) = 17.7, *P* < 0.001, because efficiency decreased as switch time increased; however, there were no interactions of switch time with the other variables (drug or initial task) *P >* 0.19. Next, by dividing the data into the first and second halves, we also assessed whether vigilance effects were present. We found results that mirrored those reported above in the primary analyses but no significant main effects or interactions regarding half, though a main effect of half for reaction time approached significance, *F*(1,308.0) = 3.20, *P* = 0.07. (This trend resulted because reaction times in the second half were slower by 17 ms, collapsed across all trial types). Additionally, to rule out the possibility that tolcapone had general effects on vigilance, subjects performed the DSST test. We did observe that the number of correct symbols completed on the DSST under bromocriptine (mean 57.1 ± standard error 3.4) was significantly fewer, *t*_(14)_ = 3.06, *P* < 0.01, than the number completed under placebo (64.3 ± 3.8), but there was no difference between tolcapone (64.5 ± 3.6) and placebo, *t*_(15)_ = 0.16, *P* = 0.44. We found also that the mean efficiency scores (collapsed across trial type) for the saccade task under each drug correlated positively with the DSST (Table 1), indicating DSST was an appropriate psychometric test for comparison to the saccade task.

**Table 1:** Mean saccade task efficiency and DSST scores

	Bromocriptine		Placebo		Tolcapone	
Subject	Efficiency	DSST	Efficiency	DSST	Efficiency	DSST
1	0.171	56	0.140	66	0.148	65
2	0.203	59	0.225	58	0.214	73
3	0.175	58	0.158	65	0.159	57
4	0.167	72	0.169	76	0.162	82
5	0.172	56	0.197	63	0.090	65
6	0.182	–	0.170	57	0.171	56
7	0.145	46	0.163	45	0.149	51
8	0.092	48	0.173	43	0.081	38
9	0.076	35	0.143	46	0.154	44
10	0.155	67	0.143	74	0.136	72
11	0.168	65	0.155	69	0.124	68
12	0.157	60	0.164	66	0.146	64
13	0.157	61	0.144	77	0.136	75
14	0.173	41	0.185	76	0.153	81
15	0.226	47	0.132	46	0.145	51
16	0.198	85	0.353	101	0.191	90
Mean	0.163	57.1	0.176	64.3	0.147	64.5
*r*	0.45		0.58^a^		0.44	
*P* value	0.096		0.018		0.091	

^a^Significant

## Discussion

In this study, human subjects performed a saccade task that required them to switch a prepared pro- or anti- saccade to the alternative on a subset of trials. We observed following the administration of tolcapone (which increases PFC dopamine tone) that subjects exhibited reduced overall performance efficiency and longer reaction times. Consistent with this finding, we also observed that subjects with the Val/Val genotype of the COMT gene (reduced PFC dopamine tone) were more efficient overall than subjects with the Val/Met or Met/Met genotypes. However, we did not observe behavioral effects following administration of bromocriptine (a D2 agonist), nor did we observe significant effects depending on response type (i.e., pro- or anti-saccade, or non-switch or switch) on either drug. The results suggest that there was a general *detrimental* influence of *greater* PFC dopamine on the ability to perform the task efficiently, as the result of tolcapone administration or of COMT genotype.

Previous studies have provided a framework for how tolcapone may improve performance by augmenting PFC dopamine tone in tasks, such as working memory tasks, which are known to be dependent on PFC function. The fact that tolcapone improved response times in working memory tasks (where information must be stored online across trials) (Apud et al. 2007; Giakoumaki et al. 2008) shows how modulation of PFC dopamine tone can have general effects on cognitive performance, rather than those limited to a response in a particular trial. Other studies on the effects of tolcapone have utilized the COMT polymorphism to show that there is an interaction between tolcapone and presumptive baseline dopamine tone, such that tolcapone improves performance in N-back working memory tasks in Val/Val individuals (who have relatively reduced PFC dopamine tone) (Apud et al. 2007; Giakoumaki et al. 2008; Cools and D’Esposito 2011).

In the present study, we observed evidence for generally worse performance under tolcapone, and we did not observe a significant COMT genotype by drug interaction (which could be the result of a lack of power). Because tolcapone increased, rather than decreased reaction times, these general effects were not likely related to dopamine acting on the motor system, which should facilitate responding as found in studies of Parkinson’s patients on dopaminergic medications (Mink 1996; Cools 2006; Cameron et al. 2012), and in one study where levodopa was provided to healthy adults (Rihet et al. 2002). (We note, however, that unlike levodopa, tolcapone is expected to have more local influences on behavior (i.e., PFC) whereas levodopa might have more widespread influences in the brain as a precursor to dopamine and norepinephrine production (Cools 2006)). Rather, our results suggest that tolcapone was detrimental to *cognitive* aspects of performance (Cools and D’Esposito 2011). Consistent with this idea, in another study using the same tolcapone dose, we also found no evidence for motor changes (Kayser et al. 2012) despite influences on reward-related decision processes.

One possible effect of tolcapone, however, is that it increased fatigue, or decreased vigilance, resulting in detriments to efficiency or reaction time. In a rat model of attention deficit hyperactivity disorder-combined type (ADHD-C), tolcapone increased sustained attention and vigilance in ADHD-C rats, but it *decreased* vigilance in healthy rats (Tomlinson et al. 2015). Lim et al. (2012) found a time-on-task effect where Met/Met subjects (greater PFC dopamine) showed greater decline in performance vigilance, but COMT polymorphism did not affect ratings of mood or fatigue (Lim et al. 2012). These results suggest that greater PFC dopamine can result in worse task performance over time, possibly related to decreased vigilance. However, in our analyses in which we divided trials into the first and second half of the task, we did not find significant evidence that tolcapone affected vigilance, nor did we find a difference between performance following tolcapone and placebo on the DSST.

Given these considerations, we propose that augmentation of PFC dopamine paradoxically improved cognitive control that was disadvantageous to performance (Diamond 2013). As in most laboratory paradigms, subjects were required to focus on the task and avoid switching their attention to irrelevant distractions or internal motivational states that would impair overall performance. This “meta-control” modulates the strength of top-down attention and cognitive flexibility, which in turn affects how easily external information gains access to working memory (Goschke and Bolte 2014). In the present study, subjects needed to prepare an instructed response to a peripheral target but at the same time monitor the fixation point for a possible change to the instructed behavior. While this necessitates focused attention, studies in performance “choking” show that *over*-focusing attention on the process of performing the task interferes with behaviors that should be more automatically driven (Kimble and Perlmuter 1970; Lewis and Linder 1997). The elevated response times supports the point that automaticity was reduced.

This proposal is supported by models on *reactive* compared to *proactive* inhibitory control (Aron 2011; Braver 2012; Schall and Godlove 2012). Reactive inhibitory control occurs in response to an exogenous stimulus (e.g., the cue to switch task) (Braver 2012). There are many possible sources of reactive inhibitory control that could be engaged by the cue: the “hyperdirect” pathway of the BG (Aron and Poldrack 2006; Aron 2011), “fixation” neurons in the frontal eye fields and superior colliculus (Munoz and Everling 2004; Schall and Godlove 2012), and substantia nigra pars reticulata (SNr) output neurons of the BG (Hikosaka et al. 2000).

In contrast, proactive inhibitory control refers to “sustained and anticipatory maintenance” of goal-relevant information, before cognitively demanding events occur (Braver 2012). In the present study, subjects had information that switches were relatively frequent (60%) and could therefore anticipate the possibility of having to switch their response. Empirical evidence suggests that this type of prediction engages proactive inhibitory control (Aron 2011), because latencies on “go” trials (where a response is required) increase when the frequency of “stop” trials increases. Likewise, we have found that when subjects move from blocked designs (pro- or anti-saccade trials separately), to ones in which trials are interleaved, to ones that include switch trials, latency increases parametrically (Cameron et al. 2010). We have also found that decreasing switch trial probability (from 75 to 50 to 25%) results in increased reaction time switch costs (Cameron et al. 2007), implying that subjects use proactive inhibitory control to modulate their behavior. Reaction times in the current study exceeded 400 ms (Fig. 3a), which is abnormally slow compared to simpler saccade tasks (Munoz and Everling 2004), but consistent with the previous studies employing this paradigm (Cameron et al. 2007, 2010). Overall, our findings support a mechanism whereby greater PFC dopaminergic tone reflects greater proactive inhibitory control.

The PFC is proposed to be the source of proactive inhibitory control signals (Braver 2012) (albeit potentially via fronto-striatal circuits (Aron 2011)), and evidence suggests that PFC-BG circuits are important when general rather than action-specific inhibition is required (Smittenaar et al. 2013). In the oculomotor system, lateral PFC is thought to be critical for suppressing a pro-saccade response (Pierrot-Deseilligny et al. 2005), by establishing anti-saccade task set signals to bias the subject against eliciting a more automatic pro-saccade *before* the stimulus appears (Everling and Johnston 2013). Increased lateral PFC activity has been observed *prior* to probe (test) stimuli in a working memory task, primarily when the expectancy of an “interference” trial was high (i.e., having to respond that a probe was *not* part of the memory set) (Burgess and Braver 2010; Braver 2012); this finding suggests that proactive inhibitory control relates to anticipatory activity in PFC, particularly in advance of more cognitively demanding tasks. Studies employing Stroop paradigms have also implicated circuits involving the lateral PFC as having a role in sustained proactive control processes when the predictability of incongruent trials is high (De Pisapia and Braver 2006; Krug and Carter 2012). Importantly, proactive inhibitory control is proposed to be dopamine dependent (Braver 2012). Accordingly, subjects with the Val allele are *less* efficient than those with the Met allele in tasks *requiring* proactive, rather than reactive inhibitory control, as subjects with the Val allele exhibit greater recruitment of lateral PFC than Met carriers (Jaspar et al. 2014).

In summary, evidence supports our proposal that increasing PFC dopamine facilitates proactive inhibitory control, which may result in an *over-focusing* of attention and thus sub-optimal performance in the saccade task. The consequence would be an “overdose” on stability, decreasing the ability to adapt to environments where *reacting* to frequent changes with behavioral modifications would be more efficient (Braver 2012).

Models of dopamine’s action on receptor subtypes support such a hypothesis. First, evidence suggests PFC neurons are more responsive to tonic dopamine signals (rather than fast-acting phasic signals); thus, increased PFC dopamine following tolcapone administration could have augmented more general attentional states (Puig et al. 2014). Second, according to the “dual-state” theory, optimal dopamine levels favor D1 receptor-mediated stabilization, but sub- or supra-optimal levels favor D2 receptor-mediated flexibility (Durstewitz and Seamans 2008; Cools 2015). This is because PFC networks are differentially affected by D1 or D2 receptor stimulation, depending on recurrent activity, such that supra-optimal D1 stimulation is detrimental because it renders the system less sensitive, and in extreme cases immune, to new information (Durstewitz and Seamans 2008). Finally, according to “inverted-U” models, increasing dopamine can move the cognitive control system from a state promoting destabilization and flexibility, to a state beneficial to stabilization and focused attention (though then further back towards a state of destabilization; Noudoost and Moore 2011; Arnsten et al. 2012; Cools 2015).

Given these relationships between dopamine’s effects on D1 and D2 receptors in PFC, we must also consider potential D2 effects from tolcapone. However, D2 effects are unlikely to fully explain our results for at least three reasons. First, reduced performance efficiency due to potential action on D2 receptors should have been evident with bromocriptine (unless the dosage was ineffective, discussed below). Second, we did not observe a *benefit* on switch trials under tolcapone (i.e., switching proactively did not actually benefit explicitly cued switch trials; Armbruster et al. 2012). Third, reaction times, and not direction accuracy, mirrored those of efficiency, showing that subjects had slower reaction times under tolcapone, rather than an increased frequency of switching errors, which might be expected from a D2-receptor effect of increased flexibility.

Finally, we hypothesized that bromocriptine would improve flexibility by acting primarily on D2 receptors in the basal ganglia (BG) (Cools et al. 2007; Surmeier et al. 2007), but we did not observe significant effects at the trial-type level or at the general level. Several studies have shown that bromocriptine influences cognitive performance, in particular in tasks requiring flexible updating of information (Kimberg et al. 1997; Cools et al. 2007; Cools and D’Esposito 2011) or in those that require resistance to distracting information (Bloemendaal et al. 2015). These effects of bromocriptine imply a mechanism of action on D2 receptors in the PFC (Cools et al. 2007; Puig et al. 2014; Bloemendaal et al. 2015) and in the BG (Cools et al. 2008). While it is possible that bromocriptine acted on D2 receptors in the PFC, PFC D2 receptors are less abundant than in the striatum (Cools 2006) and are found on output cells that project to the striatum itself (Cools and D’Esposito 2011). In any case, action of bromocriptine on D2 receptors would be expected to promote flexibility rather than stability.

It is possible that the effects of bromocriptine in the brain were reduced given that we administered the task 5 h after ingestion of the medication, based on pharmacokinetics where bromocriptine’s elimination half-life is between 3 and 7 or 8 h (Nyholm 2006; Kvernmo et al. 2006). However, we did observe a significant effect of bromocriptine in this time window (around 5 h) on DSST performance. Likewise, it is possible that the low dose (1.25 mg) that we administered was ineffective, although we have found significant behavioral effects at this dosage in other studies (Cools et al. 2007; Bloemendaal et al. 2015). Nevertheless, other studies have failed to find significant effects at this dosage with tasks performed within 3 h after ingestion (Winkel et al. 2012; Stelzel et al. 2013).

Thus, if our null finding is real, we propose that while bromocriptine likely influenced indirect pathway (D2) signals in the BG, the relative lack of agonistic action on the D1-receptor-dominated direct pathway likely suggests that flexible switching was not facilitated optimally. One extension to a standard BG model (Mink 1996) proposes that the hyperdirect pathway (a faster conduction route from cortex to the subthalamic nucleus of the indirect pathway) is important for abruptly suppressing no-longer relevant behavior (Isoda and Hikosaka 2008), but it is the *direct pathway* that is important for activating the desired behavior (Nambu 2008). This explanation is supported by recent findings by Bestmann and colleagues, who found that a high (2.5 mg) dose of the D1/D2 receptor antagonist haloperidol impaired switching to a novel motor response after subjects over-learned stimulus-response mappings in a probabilistic task (Bestmann et al. 2015). Lower doses (hypothesized to only act on D2 receptors) and the D2 receptor antagonist sulpiride did not have such effects, leading to the conclusion that motor system flexibility requires action on both the D1 and D2 receptors of the direct and indirect pathways, respectively.
